# Bioreducible Liposomes for Gene Delivery: From the Formulation to the Mechanism of Action

**DOI:** 10.1371/journal.pone.0013430

**Published:** 2010-10-15

**Authors:** Gabriele Candiani, Daniele Pezzoli, Laura Ciani, Roberto Chiesa, Sandra Ristori

**Affiliations:** 1 Department of Chemistry, Materials and Chemical Engineering “Giulio Natta”, Politecnico di Milano, Milan, Italy; 2 Chemistry Department and Center for Colloid and Surface Science (CSGI), University of Florence, Florence, Italy; Aristotle University of Thessaloniki, Greece

## Abstract

**Background:**

A promising strategy to create stimuli-responsive gene delivery systems is to exploit the redox gradient between the oxidizing extracellular milieu and the reducing cytoplasm in order to disassemble DNA/cationic lipid complexes (lipoplexes). On these premises, we previously described the synthesis of SS14 redox-sensitive *gemini* surfactant for gene delivery. Although others have attributed the beneficial effects of intracellular reducing environment to reduced glutathione (GSH), these observations cannot rule out the possible implication of the redox milieu in its whole on transfection efficiency of bioreducible transfectants leaving the determinants of DNA release largely undefined.

**Methodology/Principal Findings:**

With the aim of addressing this issue, SS14 was here formulated into binary and ternary 100 nm-extruded liposomes and the effects of the helper lipid composition and of the SS14/helper lipids molar ratio on chemical-physical and structural parameters defining transfection effectiveness were investigated. Among all formulations tested, DOPC/DOPE/SS14 at 25∶50∶25 molar ratio was the most effective in transfection studies owing to the presence of dioleoyl chains and phosphatidylethanolamine head groups in co-lipids. The increase in SS14 content up to 50% along DOPC/DOPE/SS14 liposome series yielded enhanced transfection, up to 2.7-fold higher than that of the benchmark Lipofectamine 2000, without altering cytotoxicity of the corresponding lipoplexes at charge ratio 5. Secondly, we specifically investigated the redox-dependent mechanisms of gene delivery into cells through tailored protocols of transfection in GSH-depleted and repleted *vs.* increased oxidative stress conditions. Importantly, GSH specifically induced DNA release in batch and *in vitro*.

**Conclusions/Significance:**

The presence of helper lipids carrying unsaturated dioleoyl chains and phosphatidylethanolamine head groups significantly improved transfection efficiencies of DOPC/DOPE/SS14 lipoplexes. Most importantly, this study shows that intracellular GSH levels linearly correlated with transfection efficiency while oxidative stress levels did not, highlighting for the first time the pivotal role of GSH rather than oxidative stress in its whole in transfection of bioreducible vectors.

## Introduction

Gene delivery using non-viral approaches has been extensively studied as a basic tool for intracellular gene transfer and gene therapy [1]. In the past, the primary focus has been on application of physical, chemical, and biological principles to development of a safe and efficient method that delivers a transgene into target cells for appropriate expression. Nowadays, the development of non-viral-based approaches to deliver nucleic acids to cells (transfection) is an inherently interdisciplinary endeavor and a rapidly advancing area of research [2]. Polymeric and lipidic vectors rely on the basics of supramolecular chemistry termed “self-assembling”: at physiological pH, they are cations and, after removal of small counterions, spontaneously form complexes with anionic nucleic acids [3]. Such vectors must be able to (i) complex nucleic acids in stable, nanoscaled and positively charged aggregates, (ii) promote the internalization of DNA by cells, (iii) prevent the intracellular DNA degradation and, finally, (iv) induce exogenous gene expression [4]. In this scenario, DNA/cationic lipid complexes (lipoplexes) have drawn significant attention since their use in gene therapy clinical trials is rapidly increasing (http://www.wiley.co.uk/genmed/clinical/) although their cytotoxicity and low efficiency remain major drawbacks.

Hence, in order to overcome limitations of currently available non-viral vectors, the use of stimuli-responsive carriers offer novel alternatives for the optimization of this therapy [5], [6]. Redox potential has been proposed as an efficient stimuli mechanism in gene delivery because of the high difference (10^2^–10^3^ fold) existing between the reducing intracellular space and the oxidizing extracellular milieu [7]. Indeed, the versatility of reducible disulfide carriers has been shown in many different approaches [8]–[12] but the underlying biological mechanism and physiological mediator(s) remain poorly understood [7], [13].

Since their introduction as gene carriers in 1987 [14], liposomes have become one of the most studied non-viral vectors, featuring remarkable flexibility at molecular, formulation and dimension level [15], [16]. Along this line, over the last twenty years cationic liposomes containing 1,2-dioleoyl-*sn*-glycero-3-phosphatidylcholine (DOPC) [17], [18], 1,2-dimyristoyl-*sn*-glycero-3-phosphatidylcholine (DMPC) [19], as main constituents, 1,2-dimyristoyl-*sn*-glycero-3-phosphatidylethanolamine (DMPE) [20], 1,2-dioleoyl-*sn*-glycero-3-phosphatidylethanolamine (DOPE) [17], [21], as helper lipids, and 1,2-dioleoyl-3-trimethylammonium-propane (DOTAP) [18], [21] and 3β-[*N*-(*N'*,*N'*-dimethylaminoethane)-carbamoyl]cholesterol (DC-Chol) [21] as cationic lipids have been extensively used for gene delivery purposes.

In the panorama of cationic amphiphiles, *gemini* surfactants are a relatively new class of molecules with peculiar physicochemical properties, composed by two or more head groups and two aliphatic chains, linked by a spacer [22]. Moreover, recent studies have pointed out that suitably tailored cationic *geminis* are able to yield high transfection efficiency [23]. Nevertheless, there are only a few reports on the transfection properties of *gemini* lipids [24]–[28].

**Figure 1:**
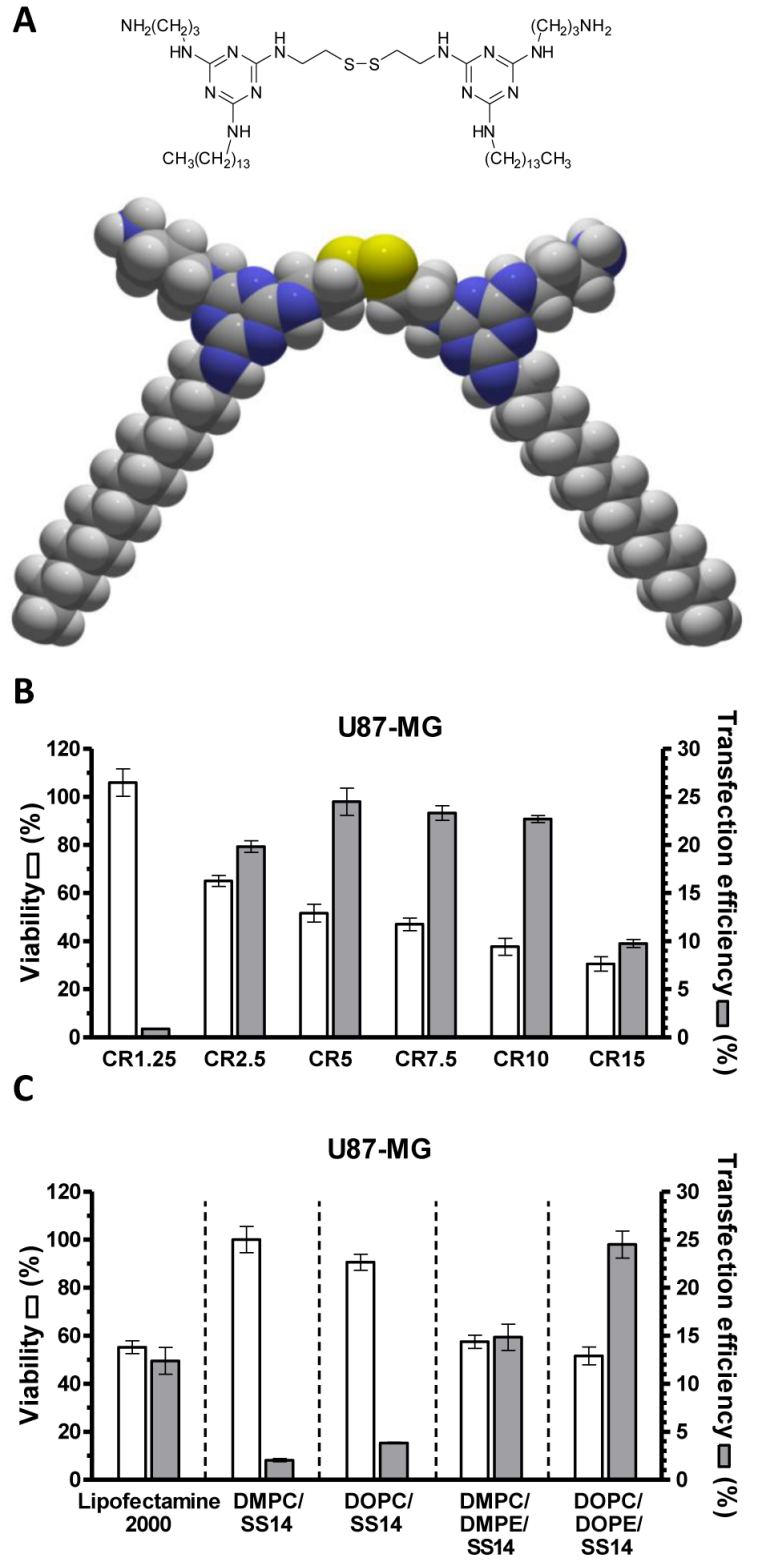
SS14 *gemini* surfactant molecule and evaluation of transfection effectiveness of SS14-containg liposome formulations. (A) Chemical structure and space-filling molecular model of *gemini* surfactant SS14. Color coding: yellow  =  sulfur; purple  =  nitrogen; grey  =  carbon; white  =  hydrogen. (B) Cytotoxicity (viability, left axis, white bars) and transfection efficiency (% of EGFP-positive cells, right axis, grey bars) of DOPC/DOPE/SS14 (25∶50∶25 molar ratio) lipoplexes on U87-MG cell line as a function of charge ratio (CR, +/−). (C) Cytotoxicity and transfection efficiency of binary DMPC/SS14, DOPC/SS14 (75∶25 molar ratio each), ternary DMPC/DMPE/SS14, and DOPC/DOPE/SS14 (25∶50∶25 molar ratio each) lipoplexes at CR5 on U87-MG cell line. Lipofectamine 2000 was used as positive control in transfection experiments. All results are expressed as mean ± SEM (n = 3).

This study ensues from our report concerning the synthesis and characterization of a new redox-sensitive triazine-based *gemini* surfactant, SS14 (Fig. 1A), for gene delivery [27]. The aim of this study was twofold. First, we studied the effects of the helper lipid composition and of the SS14 to helper lipids molar ratio on liposome dimension and overall charge (*ζ*-potential), parameters that all contribute in defining transfection efficiency and cytotoxicity. Second and most important, we sought to determine *in vitro* the physiological mechanism leading to lipoplex disassembly and gene delivery by bioreducible SS14-containing liposomes.

## Results and Discussion

### Preparation and characterization of bioreducible liposomes and lipoplexes

First, binary DOPC/SS14, DMPC/SS14 (75∶25 molar ratio each) and ternary DMPC/DMPE/SS14, DOPC/DOPE/SS14 (25∶50∶25 molar ratio each) unilamellar vesicles were designed following a number of considerations: i) the chosen co-lipids should differ both in their headgroup structure (phosphatidylethanolamine *vs.* phosphatidylcholine groups), acyl chain length and saturation degree (dimyristoyl *vs.* dioleoyl chains), to assess the effect of these components on transfection; ii) multi-component liposomes should be preferred to binary ones because of their well documented, superior transfection efficiency ; iii) SS14 content should be optimized in terms of transfection effectiveness represented by the best compromise between high transfection efficiency and low cytotoxicity.

All liposome formulations were extruded with 100 nm pore membranes. The size distribution of DOPC/SS14, DMPC/SS14, and DOPC/DOPE/SS14 liposomes was markedly narrower than that of DMPC/DMPE/SS14 formulation for which a main population with mean diameter centered at 110 nm could still be evidenced (70% by integrated intensity). On the other hand, the measured *ζ*-potential of two-component liposomes and DOPC/DOPE/SS14 formulations were, within experimental error, the same (Table 1).

**Table 1:** Hydrodynamic diameter, *ζ*-potential and polydispersity index (P.I.) of each liposome formulation.

	Liposomes		
	Diameter (nm)^a^	ζ-potential (mV)^a^	P.I.
**DMPC/SS14 (75∶25 molar ratio)**	109±3	+39±7	0.12
**DOPC/SS14 (75∶25 molar ratio)**	112±3	+40±6	0.07
**DMPC/DMPE/SS14 (25∶50∶25 molar ratio)**	110±25	+55±8	0.36
**DOPC/DOPE/SS14 (25∶50∶25 molar ratio)**	120±3	+46±8	0.07

a Mean ± Standard Deviation.

**Figure 2:**
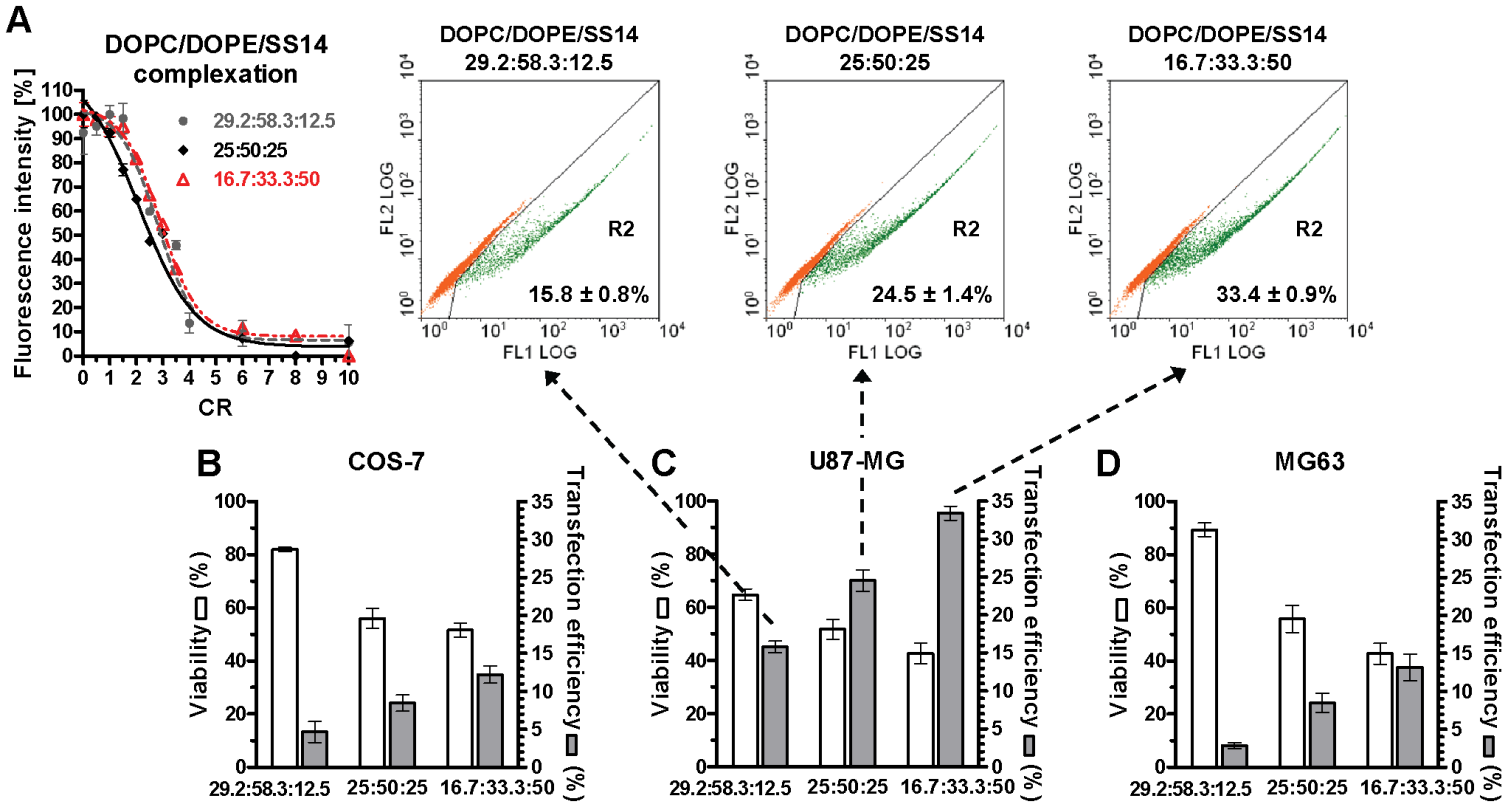
Complexation abilities of DOPC/DOPE/SS14 liposome formulations and evaluation of their transfection effectiveness. (A) Fluorophore-exclusion titration of DOPC/DOPE/SS14 liposomes at 29.2∶58.3∶12.5 (grey circles), 25∶50∶25 (black rhombus), and 16.7∶33.3∶50 molar ratios (red triangles) as a function of CR. All curves underlying data simply represent a guide to the eye and were drawn to better evidence trend variations. Cytotoxicity (viability, left axis, white bars) and transfection efficiency (% of EGFP-positive cells, right axis, grey bars) of the three different DOPC/DOPE/SS14 lipoplexes at CR5 on COS-7 (B), U87-MG (C), and MG63 (D) cell lines at CR5. Results are expressed as mean ± SEM (n = 3). Examples of cytofluorimetric analysis are reported as FL1 (green fluorescence) *vs.* FL2 (orange fluorescence) dot plot of U87-MG transfected cells (C, upper panels). Mock-transfected (pCMV-GLuc) but autofluorescent population of cells lies along the 0, 0; 10^4^, 10^4^ diagonal. EGFP-expressing cells appear as an additional population delineated by region 2 (R2), where FL1>FL2.

Based on these results all developed formulations were considered suitable candidates for further investigations as potential gene delivery vectors. We next evaluated by fluorescence titration assay the ability of all liposome formulations to complex the DNA at increasing charge ratio (CR, +/−). Interestingly, all liposomes shared the same affinity towards DNA template, represented by the lowest fluorescence values for CR≥5 (not shown). However, DNA condensation is not sufficient to ensure significant transfection levels [29]. On this ground, we decided to investigate transfection ability (evaluated as % of EGFP-positive cells) and cytotoxicity (measured by viability assay) of DOPC/DOPE/SS14 at increasing CR in U87-MG cell line commonly used in transfection experiments [30], [31]. Since use of serum cannot be avoided in long-term cultures of eukaryotic cells *in vitro* [29], transfection experiments were carried out in complete medium (D-MEM with 10% fetal bovine serum, FBS). Although this experimental condition is far from *in vivo* situation, transfections carried out in serum-complete medium are commonly used to check serum resistance of lipoplexes prior to performing animal studies [16], [31]. As expected, transfection effectiveness of lipoplexes was dramatically affected by cationic lipid to DNA ratio in that transfection efficiency followed a bell-shape trend and cell viability dramatically decreased as CR increased, as previously reported by others [32]–[34]. Among all CR tested, maximal transfection efficiency and reasonable cytotoxicity for the aims of the present work were obtained with the minimal dose of liposomes corresponding to lipoplexes at CR5 (Fig. 1B). Hence, CR5 was chosen for a comparative evaluation of all formulations. Of note, although transfection efficiencies seemed lower than for other reported transfectants [35]–[37], the method of analysis here used and firstly described by Walker and colleagues [38] allows very stringent discrimination between intrinsic autofluorescence of mock-transfected cells and truly EGFP-positive ones, as also exemplified in upper panels of Fig. 2C illustrating EGFP-positive U87-MG cells after transfection. Indeed, Lipofectamine 2000 yielded 12.4±1.4% of EGFP-positive U87-MG cells (Fig. 1C), similar or lower than transfection efficiencies observed with DMPC/DMPE/SS14 and DOPC/DOPE/SS14 formulations, using this analytical method.

In DOPC/SS14 lipoplexes, the presence of unsaturated acyl dioleoyl chains conferred higher transfection efficiency compared to saturated dimyristoyl chains (3.8±0.1% *vs.* 2.0±0.2%, *p*<0.05) with no appreciable difference in cytotoxicity (viability: 90.6±3.3% *vs.* 100.1±5.4%, not statistically significant). Our results are in agreement with Felgner *et al.* that firstly showed that the transfection efficacies in a series of homologous lipids with symmetric saturated hydrophobic moieties were C_oleyl_>C_16_>C_18_>C_14_ [39]. In parallel, the introduction in our liposome formulations of helper lipids bearing phosphatidylethanolamine polar heads increased transfection efficiency up to 7.4- and 6.5-fold in DMPC/DMPE/SS14 and DOPC/DOPE/SS14 lipoplexes, with respect to each binary counterpart. These results highlight the superior transfection efficiency of multicomponent lipoplexes with respect to binary ones, as previously reported by Caracciolo and colleagues [40]. Although some speculative arguments have been proposed, the exact physico-chemical reasons why multicomponent lipoplexes are more efficient than binary lipoplexes have never been stated unambiguously [41]. On the other hand, viability of cells transfected with phosphatidylethanolamine-containing lipoplexes decreased by 1.7- and 1.9-fold, respectively. The strong transfection efficiency-dependence on DOPE and DMPE presence in liposome formulations supports the role of the phosphatidylethanolamine headgroup as membrane fusion or destabilization agent due to its ability to promote transition from the bilayer phase (L_α_) into the inverted hexagonal phase (H_II_) [39], [42]. Altogether, DOPC/DOPE/SS14 represented the best compromise between the highest transfection efficiency (24.5±1.4% *vs.* 14.8±1.3% for DMPC/DMPE/SS14, *p*<0.05) and acceptable cytotoxicity (viability: 51.7±3.7% *vs.* 57.5±2.8 for DMPC/DMPE/SS14, not statistically significant) (Fig. 1c). Cytotoxicities were in line with those reported by others with DOPE-containing liposomes evaluated 48 h post-transfection [43] and comparable to that of the gold standard Lipofectamine 2000 (Viability: 55.2±2.7%), as reported in Fig. 1C. Noteworthy, our DOPC/DOPE/SS14 lipoplex formulation yielded almost two-fold improved transfection efficiency compared to Lipofectamine 2000 (24.5±1.4% *vs.* 12.4±1.4% for Lipofectamine 2000, *p*<0.05).

Thus, we focused on developing and optimizing the DOPC/DOPE/SS14 liposome formulation with the aim of determining its specific mechanism of transfection. In order to find the optimal SS14 ratio for transfection experiments, three DOPC/DOPE/SS14 formulations at different SS14 molar fractions (29.2∶58.3∶12.5, 25∶50∶25, and 16.7∶33.3∶50 molar ratios) were prepared. Next, chemical-physical properties and the corresponding transfection efficiencies were examined (Fig. 2).

As reported above, an asymptotic decrease in fluorescence was observed for all liposome formulations at CR5 (Fig. 2A), which still represented the best compromise between high transfection efficiency and low cytotoxicity *in vitro* (not shown). Therefore, we evaluated both size and *ζ*-potential for all liposomes and for corresponding lipoplexes at CR5 (Table 2).

**Table 2:** Hydrodynamic diameter, *ζ*-potential and polydispersity index (P.I.) of DOPC/DOPE/SS14 liposomes and lipoplexes at CR5.

	Liposomes			Lipoplexes at CR5		
	Diameter (nm)^a^	ζ-potential (mV)^a^	P.I.	Diameter (nm)^a^	ζ-potential (mV)^a^	P.I.
**DOPC/DOPE/SS14 (29.2∶58.3∶12.5 molar ratio)**	134±4	+44±9	0.05	295±12	+20±4	0.32
**DOPC/DOPE/SS14 (25∶50∶25 molar ratio)**	120±3	+46±8	0.07	259±3	+30±3	0.04
**DOPC/DOPE/SS14 (16.7∶33.3∶50 molar ratio)**	129±3	+50±9	0.03	291±7	+26±4	0.09

a Mean ± Standard Deviation.

Liposomes extruded at 100 nm had a hydrodynamic diameter of circa 130 nm and a *ζ*-potential ranging from +44 to +50 mV. As expected, after complexation with DNA at CR5, the hydrodynamic diameter increased on average by 2.2-fold and the overall charge decreased by almost twofold. Since transfection effectiveness depends to a great extent on the cell type and the lipid composition [29], [41], we tested DOPC/DOPE/SS14 formulations on three different cell lines. In spite of similar homogeneities in size and *ζ*-potential, DOPC/DOPE/SS14 lipoplexes with the highest SS14 content (16.7∶33.3∶50 molar ratio) showed the highest transfection efficiency in all cell lines tested (*p*<0.05), reaching up to circa 35% of EGFP-positive U87-MG cells (Fig. 2C upper panels), while the cytotoxicity was similar to that of DOPC/DOPE/SS14 at 25∶50∶25 molar ratio (not statistically significant) (Fig. 2B–D). Noteworthy, in U87-MG cell line the transfection efficiency of DOPC/DOPE/SS14 liposomes at 29.2∶58.3∶12.5, 25∶50∶25, and 16.7∶33.3∶50 molar ratios, were 1.3-, 1.9-, and 2.7-fold higher than that of the benchmark Lipofectamine 2000, as shown in Fig. 1C and 2C. In agreement with a previous report [41], we found that both the number of lipid components and their relative molar ratio in multicomponent liposomes altered transfection activity. In particular, transfection efficiency of DOPC/DOPE/SS14 lipoplexes peaked at 16.7∶33.3∶50 molar ratio when both SS14 cationic and neutral lipid species were mixed in equimolar ratio.

Hence, neither size nor *ζ*-potential of the complexes was clearly associated with transfection efficiency while lipid composition and relative molar ratios affected it. In agreement with our findings, Farhood and colleagues previously reported that, in cationic DOPE/DC-Chol liposome formulations, transfection efficiency was proportional to DC-Chol content, with the optimum at 50–60% of cationic lipid [44]. Moreover, in accordance with our results, Pinnaduwage *et al.* showed, in three different DOPE-containing liposomes, that cytotoxicity was directly related to the amount of the cationic component [45].

### Effect of GSH on bioreducible lipoplexes

**Figure 3:**
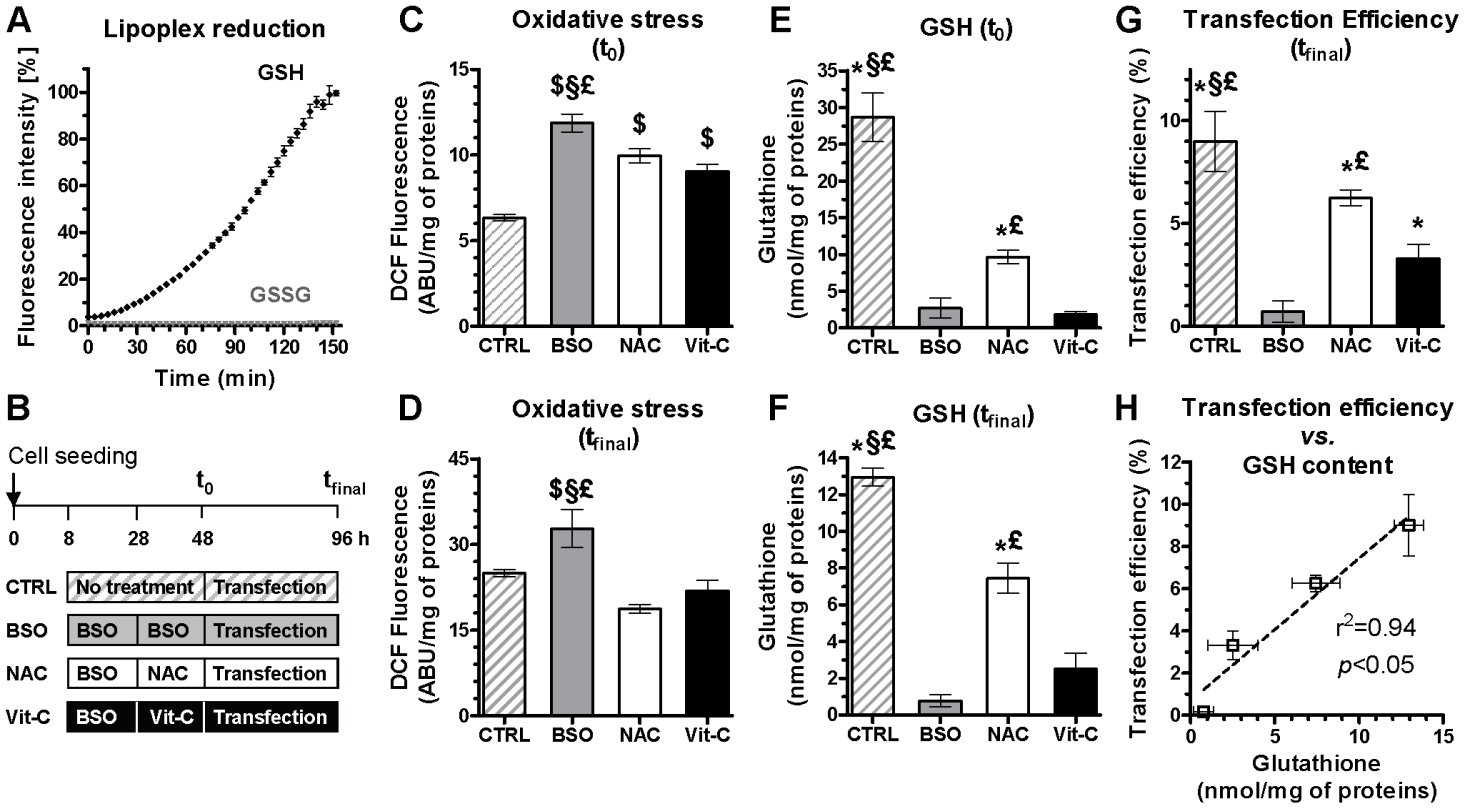
GSH-mediated lipoplex disassembly in batch and effect of intracellular GSH levels on transfection efficiency. (A) Stability of DOPC/DOPE/SS14 (16.7∶33.3∶50 molar ratio) lipoplexes at CR5 in presence of GSH or GSSG. Results are presented as % of fluorescence emitted with respect to DNA. (B) Experimental procedure. MG63 cells were divided in four groups: untreated CTRL, BSO-, NAC-, and Vit-C-treated cells. Following pharmacological treatment (t_0_), cells underwent 48 h transfection (t_final_) with DOPC/DOPE/SS14 (16.7∶33.3∶50 molar ratio) lipoplexes at CR5. Oxidative stress and GSH content were measured at t_0_ ((C) and (E), respectively) and after transfection ((D) and (F), respectively). Transfection efficiency, expressed as % of EGFP-positive cells, was also evaluated (G). A linear correlation between GSH content and transfection efficiency was observed (H). Results are expressed as mean ± SEM (n = 3). $ *p*<0.05 *vs.* CTRL; * *p*<0.05 *vs.* BSO; § *p*<0.05 *vs.* NAC; £ *p*<0.05 *vs.* Vit-C.

An important feature of our liposome formulations is the disulfide linker moiety in SS14 that, in suitable reducing environment, might promote lipoplex disassembly by reversion of the *gemini* surfactant to single-chain amphiphiles. According to the existing literature, the intracellular reduction of disulfide bonds in lipo/polyplexes is most likely mediated by small redox molecules [46]. Among antioxidants, glutathione (L-γ-glutamyl-L-cysteinyl-glycine) is the most abundant non-protein thiol in mammalian cells, typically present in the reduced form (GSH) and oxidized glutathione disulfide (GSSG) [47], with an overall cellular GSH/GSSG ratio ranging from 30∶1 to 100∶1 [48]. Although glutathione is ubiquitous, it is present in high levels (1–11 mM) intracellularly and at low concentration (10 µM) in the extracellular milieu [13]. Since the increasing content of redox-sensitive surfactant, SS14, along DOPC/DOPE/SS14 series correlated with higher transfection efficiency, we next examined by fluorescence titration assay whether GSH might lead to lipoplex disassembly and DNA release. As shown in Fig. 3A, in test tube, only reducing GSH triggered DNA release from DOPC/DOPE/SS14 (16.7∶33.3∶50 molar ratio) lipoplexes.

Although others have conferred the beneficial effects of intracellular reducing environment to glutathione only [46], [49], [50], the possible implication of the reducing milieu in its whole on transfection efficiency of bioreducible transfectants has been overlooked. In particular, the thioredoxin (Trx) system, composed of thioredoxin reductases (TrxR), thioredoxins and NADPH [51] is known to participate in modulating the intracellular redox environment and thiol/disulfide exchange, rendering difficult to single out the effect of GSH *per se* in the context of the overall redox state [52]. Most studies have relied on the use of the glutathione depletor L-buthionine-sulfoximine (BSO) to demonstrate the link between glutathione content and transfection efficiency of bioreducible transfectants. However, although BSO does modulate glutathione synthesis inhibiting γ-glutamylcysteine synthetase [53], it has also been described to alter expression profiles of several genes involved in redox homeostasis [54], [55]. As a consequence, the unique role of glutathione in physiological disulfide-containing lipoplex reduction and disassembly has never been strikingly demonstrated. For this reason we here evaluated the contribution of both the overall redox status as well as GSH only on transfection efficiency.

In this context, we studied how the intracellular redox status and GSH content separately modulate the transfection activity of DOPC/DOPE/SS14 (16.7∶33.3∶50 molar ratio) bioreducible lipoplexes. To this end, MG63 cells were supplemented with the GSH depletor BSO for 20 h, after which cells were treated with either BSO, the glutathione repletor *N*-acetyl-L-cysteine (NAC) or the antioxidant L-ascorbic acid (Vitamin C, Vit-C) for another 20 h before transfection (t_0_) (Fig. 3B). At t_0_ BSO treatment increased by almost twofold oxidative stress levels with respect to untreated cells (CTRL) (*p*<0.05), while the antioxidant treatment with NAC and Vit-C equally alleviated BSO effects (Fig. 3C).

Noteworthy, antioxidants cannot indiscriminately be lumped together. Although Vit-C is part of an antioxidant network where GSH plays a pivotal role, recycling other antioxidants and keeping them in their active state, it does not compensate for GSH depletion [56]–[58]. In this regard, a few studies supported the use of supplemental Vit-C in individuals predisposed to reduced GSH levels, either due to age [59] or diseases [60], [61]. Although dietary supplementation with Vit-C restored resistance to oxidative stress and its sequelae, it did not replenish GSH levels [62]. Indeed, in our study only preincubation of GSH-depleted cells with NAC partially restored GSH levels (t_0_; 9.7±0.9 *vs.* 2.7±1.3 and 1.9±0.4 nmol/mg of proteins for BSO and Vit-C groups, respectively, *p*<0.05) (Fig. 3E). Noteworthy, both NAC- and Vit-C-treated cells shared the same oxidative stress levels (not statistically significant) (Fig. 3C) significantly lower than those of BSO group (t_0_; 11.9±0.5 *vs.* 9.9±0.4 and 9.1±0.4 ABU/mg of proteins for NAC and Vit-C groups, respectively, *p*<0.05), highlighting the unspecific antioxidant effects of both. Afterwards, cells underwent 48 h transfection (t_final_). 9.0±1.5% of EGFP-positive cells were observed in the CTRL group compared to 6.3±0.4% in NAC- and 3.3±0.7% in Vit-C-treated groups (Fig. 3G). Remarkably, after transfection, oxidative stress levels in NAC- and Vit-C-treated groups were equal to CTRL (not statistically significant) (Fig. 3D), as previously shown by Shang and co-workers in an *in vitro* study dealing with effects of GSH and Vit-C in BSO-treated epithelial cells [58]. However, only the NAC-treated group showed a significantly high, 57% repletion in GSH content compared to BSO-untreated CTRL (t_final_; 7.4±0.8 *vs.* 0.8±0.3 and 2.5±0.8 nmol/mg of proteins for BSO and Vit-C groups, respectively, *p*<0.05) (Fig. 3F) yielding improved transfection efficiency (*p*<0.05 *vs.* BSO and Vit-C) (Fig. 3G). Finally we found a linear correlation between GSH content and transfection efficiency (r^2^ = 0.94, *p*<0.05) in cells transfected with bioreducible DOPC/DOPE/SS14 (16.7∶33.3∶50 molar ratio)-based lipoplexes (Fig. 3H). Inversely, oxidative stress levels and transfection efficiency did not correlate at all (r^2^ = 0.35, *p* = 0.21, not statistically significant). As a proof of concept, transfection efficiency of non-reducible transfectant Lipofectamine 2000 did not rely on either oxidative stress status (r^2^ = 0.37, *p* = 0.39, not statistically significant) or GSH content (r^2^ = 0.39, *p* = 0.38, not statistically significant), specifically attributing the above demonstrated crucial role of GSH to bioreducible transfectants.

In summary, we have developed novel effective ternary DOPC/DOPE/SS14 liposomes for gene delivery formulated with the bioreducible cationic *gemini* surfactant, SS14, previously synthesized by our group [27]. In such formulations, the presence of unsaturated acyl (dioleoyl) chains and phosphatidylethanolamine polar heads conferred superior transfection efficiency to the corresponding lipoplexes. By raising the SS14 content in DOPC/DOPE/SS14 liposome series up to 50% (mol/mol), transfection efficiency increased, overreaching by almost three-fold the transfection efficiency observed with the commercially available Lipofectamine 2000 (33.4±0.9% *vs.* 12.4±1.4% for Lipofectamine 2000, *p*<0.05). Moreover, we demonstrated that physiological concentration of GSH could mediate DOPC/DOPE/SS14 (16.7∶33.3∶50 molar ratio) lipoplex disruption and DNA release induced by SS14 reduction in batch. Finally, the major finding of this work regards the linear correlation between GSH content and transfection efficiency in cells transfected with these lipoplexes, underscoring the fundamental role of GSH levels in modulating transfection effectiveness of bioreducible lipoplexes.

Overall this work underlines for the first time the pivotal role of high intracellular GSH content in gene delivery efficiency of bioreducible carriers, opening the door towards an improved development of these and other bioreducible transfectants as potential therapeutics of the future in particular disease contexts, as increased levels of intracellular GSH have been associated with anticancer drug resistance [63]–[65] and xenobiotic liver detoxification [66]. Since new bioreducible SS14-based transfectants seem interesting for evaluating the effect of innate glutathione levels on transfection efficiency of bioreducible transfectants, *in vivo* effectiveness of DOPC/DOPE/SS14 lipoplexes will be investigated in the future.

## Materials and Methods

### Materials

Plasmid DNA encoding for the Enhanced Green Fluorescent Protein (pEGFP-N1) or for the secreted Gaussia Luciferase (pCMV-GLuc) were purchased from Clontech Laboratories (Mountain View, CA, USA) and from New England BioLabs (Hitchin, UK), respectively. DOPC, DMPC, DMPE, and DOPE were from Avanti Polar Lipids (Alabaster, AL, USA). Lipofectamine 2000 was from Invitrogen Life Technologies (San Giuliano Milanese, Italy). All chemicals were from Sigma-Aldrich (Milan, Italy) if not differently specified. SS14 *gemini* surfactant was previously synthesized by our group [27]. U87-MG (human glioblastoma-astrocytoma, epithelial-like cell line, HTB-14), MG63 (human osteosarcoma cell line, CRL-1427) and COS-7 (African green monkey kidney fibroblast-like cell line, CRL-1651) were purchased from the European Collection of Cell Cultures (ECACC, Salisbury, UK).

### Liposome preparation

Stock solutions of liposomes were prepared from binary mixtures of DOPC/SS14 and DMPC/SS14 at 75∶25 molar ratio each and from ternary mixtures of DMPC/DMPE/SS14 at 25∶50∶25 molar ratio, and DOPC/DOPE/SS14 at 29.2∶58.3∶12.5, 25∶50∶25, and 16.7∶33.3∶50 molar ratios, with a final concentration of helper lipids of 14 mM. Mixtures of dry lipid powders were dissolved in chloroform and after solvent evaporation the lipid film was swollen at room temperature (r.t.) with deionized water. Multilamellar vesicles obtained upon vortexing were then submitted to eight freeze/thaw cycles and extruded through 100 nm-pore polycarbonate membranes (27 passages; LiposoFast apparatus, Avestin, Ottawa, Canada) to obtain monodisperse small monolamellar vesicles. Samples were stored at 4°C.

### Lipoplex formation and disruption

Each lipoplex sample was prepared at r.t. by adding a solution of nucleic acids (pEGFP-N1) to a liposome suspension, at the desired lipid concentration, yielding different CR. The DNA binding ability of each liposome formulation was monitored by a fluorophore-exclusion titration assay. For each condition, 0.12 µg of pEGFP-N1 in 2.4 µl of SYBR Green I (λ_ex_ = 497 nm; λ_em_ = 520 nm) were added to 3.6 µl of liposome suspension at different concentrations in order to achieve the desired CR. The fluorescence of the intercalated dye was measured using GENios Plus reader (Tecan, Segrate, Italy) in black 384-well microplates. The effect of reduction on lipoplex stability was examined on DOPC/DOPE/SS14 (16.6∶33.3∶50 molar ratio) complexed at CR5 by measuring the ability of GSH to restore the fluorescence of DNA/SYBR Green I. Five µl of lipoplexes containing 0.1 µg of pEGFP-N1 in SYBR Green I solution were diluted 1∶20 in 10 mM aqueous solution of either GSH or GSSG. Lipoplex reduction was monitored at 37°C by measuring the fluorescence emitted as described above.

### Liposome and lipoplex dimensions and overall charge

The size of liposomes and the corresponding lipoplexes were determined by Dynamic Light Scattering (DLS) with a Malvern ZS ZEN3500 particle sizer. In this apparatus the light scattered by a laser of 532 nm wavelength, is detected at 173° with respect to the incoming ray (Back Scattering technique), amplified and then analyzed by a correlator. The obtained correlation function, whose shape depends on the size of the scattering objects, was analyzed by a procedure based on the algorithm CONTIN, which gives mean diameter and polydispersity index of the liposome size distribution. The ζ-potential was obtained by Laser Doppler Velocimetry in the same Malvern ZS ZEN3500 apparatus. In this case, the electrophoretic mobility was measured with Phase Analysis Light Scattering (PALS) technique, from which the ζ-potential is extracted by using the Smoluchowsky equation. All liposome and lipoplex suspensions were diluted 1∶20 for both size and ζ-potential measurements, in order to meet the best sensitivity requirements.

### Transfection experiments

U87-MG, MG63, and COS-7 cell lines were cultured at 37°C in a humidified atmosphere of 5% CO_2_, with complete medium consisting in Dulbecco's Modified Eagle Medium (D-MEM) with 10% (v/v) FBS, 1 mM sodium pyruvate, 10 mM Hepes buffer, 100 U/ml penicillin, 0.1 mg/ml streptomycin, and 2 mM glutamine. Before experiments, 1×10^4^ cells/cm^2^ were plated in 12-well cell culture plates and allowed to adhere overnight. The day of transfection, cells were washed once in phosphate-buffered saline (PBS) and the culture medium was replaced with 800 µl/well of complete medium containing lipoplexes (80 ng/cm^2^ of pDNA) at the desired CR and transfected for 48 h. Transfection experiments with Lipofectamine 2000 were carried in Opti-MEM according to the manufacturer's procedures, utilizing 80 ng/cm^2^ of pEGFP-N1 to easily compare results among transfectants. The cells were trypsinized, fixed in 4% (w/v) paraformaldehyde (PFA) in PBS and stored at 4°C. Transfection efficiency was measured evaluating the percentage of EGFP-positive cells in each sample by means of a flow cytometer (FACS-Calibur, Becton Dickinson, Buccinasco, Italy); a minimum of 1×10^4^ cells was analyzed for each sample. EGFP was excited at 488 nm and emitted light was collected at 520 nm (green fluorescence) and 575 nm (orange fluorescence) to enable correction for autofluorescence by diagonal gating [11]. Background fluorescence and autofluorescence were determined using mock-transfected cells (pCMV-GLuc) and subtracted to EGFP-positive cells. Cellular debris showing reduced side scattering (SSD) and forward scattering (FSD) were excluded from analysis. Data were analyzed by WinMDI2.9 software program and transfection efficiency was expressed as the percentage of EGFP-positive cells over the total cell number. Cytotoxicity of lipoplexes was tested using AlamarBlue cell viability assay (Invitrogen Life Technologies, San Giuliano Milanese, Italy) according to manufacturer's guidelines. Viability of untreated control cells was assigned as 100%.

### GSH depletion/repletion

For GSH depletion/repletion study, MG63 cells were plated in T25 flasks at a density of 1×10^4^ cells/cm^2^ in complete medium. Eight hours after plating, cell culture medium was supplemented with 0.05 mM BSO. After 20 h incubation, cells were washed in PBS and the medium was replaced with fresh complete medium supplemented either with 0.05 mM BSO, 1 mM NAC, or 0.2 mM Vit-C for further 20 h (t_0_). Finally, the medium was replaced with complete medium containing either DOPC/DOPE/SS14 (16.6∶33.3∶50 molar ratio) liposomes corresponding to CR5 or 0.16 µl of Lipofectamine 2000 complexed with 80 ng/cm^2^ of pEGFP-N1 and cells were transfected for 48 h (t_final_). Mock-transfected cells were done in parallel. At t_0_ and t_final_ cells were trypsinized, harvested and each sample was splitted into two aliquots. In one-half aliquots, cell rupture was achieved by five pulses (1 min each, 20 kHz, 100 W) on Labsonic L sonicator (B Braun, Melsungen, Germany), alternated by 1 min intervals on ice and the cell debris were removed by centrifugation (1×10^4^ rpm for 10 min). Protein content in cell lysate was determined by BCA protein assay kit (Pierce, Rockford, IL, USA). After the addition of 5% (w/v) sulfosalicylic acid (SSA), GSH levels were determined with Glutathione assay kit (Sigma-Aldrich, Milan, Italy) according to the manufacturer's instructions, except that Glutathione Reductase and NADPH were not added to samples. In the remaining half-aliquots, cells were counted (trypan blue staining), fixed, and transfection efficiency was evaluated by means of a flow cytometer. Oxidative stress was monitored by 2′,7′-dichlorodihydrofluorescein diacetate (DCFH-DA) assay [67]. Briefly, at the desired time point, cells were washed in PBS, incubated for 15 min at 37°C with 10 µM DCFH-DA in PBS and washed twice with PBS and lysed with 50 mM Tris-HCl buffer pH 7.5, containing 0.5% (v/v) Tween 20. Finally, cells were detached and centrifuged for 5 min at 1×10^4^ rpm to remove cell debris. Intracellular de-esterification and oxidation of DCFH-DA produce the highly fluorescent 2′,7′-dichlorofluorescein (DCF). DCF fluorescence (λ_ex_ = 485 nm; λ_em_ = 530 nm) was measured using GENios Plus reader. Fluorescence results were normalized over protein content of each sample.

### Statistical analysis

Statistical analysis was carried out by GraphPad version 5.0 (GraphPad software, La Jolla, CA, USA). Comparisons among groups were performed by one-way ANOVA, with Bonferroni's Multiple Comparison Test and correlations were analyzed by Pearson Test. Significance was retained when *p*<0.05.
